# Retinal Thickness and Microvascular Pattern in Early Parkinson's Disease

**DOI:** 10.3389/fneur.2020.533375

**Published:** 2020-10-07

**Authors:** Cristina Rascunà, Andrea Russo, Claudio Terravecchia, Niccolò Castellino, Teresio Avitabile, Vincenza Bonfiglio, Matteo Fallico, Clara Grazia Chisari, Calogero Edoardo Cicero, Marco Grillo, Antonio Longo, Antonina Luca, Giovanni Mostile, Mario Zappia, Michele Reibaldi, Alessandra Nicoletti

**Affiliations:** ^1^Section of Neurosciences, Department of Medical, Surgical Sciences and Advanced Technologies GF Ingrassia, University of Catania, Catania, Italy; ^2^Department of Ophthalmology, University of Catania, Catania, Italy

**Keywords:** parkinson's disease, retina, vascularization, optical coherence tomography, optical coherence tomography angiography

## Abstract

A thinning of intraretinal layers has been previously described in Parkinson's disease (PD) patients compared to healthy controls (HCs). Few studies evaluated the possible correlation between retinal thickness and retinal microvascularization. Thus, here we assessed the thickness of retinal layers and microvascular pattern in early PD patients and HCs, using, respectively, spectral-domain optical coherence tomography (SD-OCT) and SD-OCT-angiography (SD-OCT-A), and more interestingly, we evaluated a possible correlation between retinal thickness and microvascular pattern. Patients fulfilling criteria for clinically established/clinically probable PD and HCs were enrolled. Exclusion criteria were any ocular, retinal, and systemic disease impairing the visual system. Retinal vascularization was analyzed using SD-OCT-A, and retinal layer thickness was assessed using SD-OCT. Forty-one eyes from 21 PD patients and 33 eyes from 17 HCs were evaluated. Peripapillary retinal nerve fiber layer (RNFL) and macular RNFL, ganglionic cell layer (GCL), inner plexiform layer (IPL), and inner nuclear layer (INL), resulted to be thinner in PD compared to HCs. Among PD patients, a positive correlation between RNFL, GCL, and IPL thickness and microvascular density was found in the foveal region, also adjusting by age, sex, and, especially, hypertension. Such findings were already present in the early stage of disease and were irrespective of dopaminergic treatment. Thus, the retina might be considered a biomarker of PD and could be a useful instrument for onset and disease progression.

## Introduction

Parkinson's disease (PD) is the second most common neurodegenerative disease after Alzheimer's disease (AD), affecting up to 4% of the population in the oldest age groups. PD is traditionally related to a progressive loss of dopaminergic neurons in the substantia nigra pars compacta, leading to motor symptoms, such as bradykinesia, resting tremor, and rigidity (1). Moreover, PD patients may experience non-motor symptoms such as mood dysfunction, personality disorders (2, 3), autonomic failure (4), cognitive impairment (5), sleep disorders (6), and visual disturbances (7), probably related to alpha-synuclein (αSYN) inclusions in both central and peripheral nervous systems (8, 9). While motor symptoms occur when at least 50–60% of dopaminergic neurons have been lost, non-motor symptoms may occur many years before onset of motor manifestations, playing an intriguing role as biomarker in the diagnosis of “Prodromal PD” (10). Among non-motor symptoms, visual disorders are extremely common, also in the early stage of disease, affecting up to 78% of patients with PD (7). Ocular symptoms include impairment of color vision (11, 12), contrast sensitivity (7, 11–14), and visual acuity (15, 16). Several optical coherence tomography (OCT) studies showed a thinning of intraretinal layers in PD patients compared to HCs, especially in retinal nerve fiber layer (RNFL), ganglionic cell layer (GCL), and inner plexiform layer (IPL) (17–20). Recently, also microvascular retinal density has been described to be lower in PD patients compared to HCs, using spectral-domain OCT angiography (SD-OCT-A) (21).

Thus, the aims of our study were to compare the thickness of retinal layers between PD patients and HCs using SD-OCT and to evaluate their possible correlates with microvascular pattern using SD-OCT-A.

## Materials and Methods

### Study Population

Subjects attending at “Parkinson's Disease and Movement Disorders Center” of the University of Catania who fulfilled the MDS-PD diagnostic criteria for clinically established or clinically probable PD (22) were enrolled. Data about disease onset, clinical characteristics, disease duration, dopaminergic treatment, and disease severity were collected.

A group of HCs without PD or any other neurodegenerative disease was also enrolled in the study.

For both PD and HC subjects, exclusion criteria were a history of ocular trauma, previous ocular surgery that could impair the visual pathway or macular morphology, concurrent ocular diseases (including retina, optic nerve, cornea, and macular diseases), intraocular pressure (IOP) > 21 mmHg, media opacifications, systemic conditions that could impair the visual system, such as diabetes mellitus, uncontrolled hypertension or hypotension, cardiovascular diseases, and any other neurological disease.

All the subjects underwent a complete neurological examination by a neurologist expert in movement disorders, including the administration of Unified Parkinson's Disease Rating Scale part III (UPDRS-III) (23), Hoehn and Yahr (HY) scale (24), and Montreal Cognitive Assessment (MoCA) (25). All subjects were screened for subjective visual disturbances using validated questionnaires for visual acuity (26), color discrimination (27), and stereopsis (28). Moreover, all subjects underwent a complete ophthalmologic examination at “Ophthalmology Clinic” of the University of Catania, including visual acuity and IOP evaluation, fundus examination, slit-lamp biomicroscopy, SD-OCT, and SD-OCT-A assessment.

### High-Definition Optical Coherence Tomography (HD-OCT) Imaging

Macular retinal thickness and peripapillary retinal nerve fiber layer (RNFL) thickness were assessed using the Cirrus HD-OCT model 5000 (Carl Zeiss Meditec, Inc).

To examine the macula, the Macular Cube 512 × 128 protocol was used. The macular cube 512 × 128 scan uses a raster scan mode that scans a 6 × 6-mm macular area into 512 × 128 (length by width) points. Layer segmentation of the OCT data was performed using a previously developed and validated algorithm for detecting seven layers within the macula. The algorithm had three stages: pre-processing, pixel classification, and graph-based multilayer segmentation. Additionally, estimates of the inner and outer retinal boundaries (inner limiting membrane and Bruch's membrane) were used to restrict the region of interest for the algorithm, as well as to flatten the data to the Bruch's membrane boundary. Constraints were used to limit the minimum and maximum distance between each boundary and to limit the smoothness of the final segmentation. Moving from the inside to the outside, the retina resulted to be composed by the following layers: RNFL, ganglionic cell layer (GCL), inner plexiform layer (IPL), inner nuclear layer (INL), outer plexiform layer (OPL), outer nuclear layer (ONL), and retinal pigment epithelium (RPE). The average peripapillary RNFL thickness and the RNFL thickness along the superior, temporal, inferior, and nasal sectors of optic nerve were also assessed. Peripapillary RNFL thickness was acquired with the Optic Disc Cube 200 × 200 protocol that images the optic disc in a 6 × 6-mm region.

### Spectral-Domain Optical Coherence Tomography Angiography (SD-OCT-A) Imaging

SD-OCT-A of the macula and peripapillary plexus was performed employing AngioVue XR Avanti (Optovue Inc, Fremont, California, USA). The A-scan rate was of 70,000 per second, and a light source centered on 840 nm was used. The acquired OCT-A images of the macula (3 × 3 mm) were centered on the foveola. Each OCTA en-face image contains 304 × 304 pixels derived from the intersection of the 304 vertical and 304 horizontal B-scans. AngioVue automatically segments the area into four layers, including superficial capillary plexus layer (SCP), deep capillary plexus layer (DCP), outer retina layer, and choriocapillaries. In addition, the analyses of vascular density in SCP and DCP were focused on subsectors of the whole area including fovea, parafoveal area, and two hemispheres, namely, Superior and Inferior, divided by a horizontal line through the foveal center.

OCT-A of the peripapillary plexus was acquired by 4.5 × 4.5-mm images centered on the optic nerve head (ONH). Vascular density (%) was calculated automatically for the whole en-face image and divided in the hemi-superior and hemi-inferior sectors. The analysis provides data of vascular density about the optic disc only (ONH) and for the peripapillary region. Eight subsectors of the peripapillary area were also calculated including the nasal-superior (NS), nasal-inferior (NI), inferior-nasal (IN), inferior-temporal (IT), temporal-inferior (TI), temporal-superior (TS), superior-temporal (ST), and superior-nasal (SN) subsectors. Quantitative analyses of vascular density (%) in superficial, deep capillary plexus, peripapillary plexus, and analysis of fovea avascular zone (FAZ) area (mm^2^) were performed by AngioAnalytics software (Optovue Inc., Fremont, CA, USA) provided by the machine.

### Data Collection

For each subject, ocular examination was performed on left and right eye and data from both eyes were considered to perform statistical analysis. The quality of each ocular image was evaluated by an expert in ophthalmology, and eyes whose ocular measurements were judged of poor quality were excluded from the analysis. Collected data has been coded, and subjects have been assigned a unique identification code to protect anonymity. Data has been entered in an *ad hoc* created database using Excel software. Before analysis, a consistency check has been performed for all the variables in the database.

### Statistical Analysis

Data were analyzed using STATA 12.1 software packages. Quantitative variables were described using mean and standard deviation. The difference between means was performed using *t*-test. The difference between proportions was assessed using the chi-square test.

To evaluate the possible association between PD and thickness of each retinal layers, an unconditional logistic regression analysis was performed by considering the presence of PD as the outcome variable. The odds ratios (OR) with 95% confidence intervals (CI) and *p*-value (two-tailed test, *a* = 0.05) were calculated. Parameters associated with the outcome at the univariate analysis with a threshold of *p* = 0.10 were included in the model. For each retinal layer, the multivariate model was constructed considering age and sex as *a priori* confounders. Moreover, considering the influence of blood pressure values on microvascular pattern, all results were adjusted also by presence/absence of hypertension on medical history, using the multivariate model.

Pearson correlation analysis was performed to evaluate the presence of correlation between retinal layers thickness and microvascular pattern. These data were also adjusted for age, sex, and hypertension. The significance level was set at 0.05, and the 95 confidence intervals (95% CI) were calculated.

### Standard Protocol Approvals, Registrations, and Patient Consents

The study was conducted in accordance with the Declaration of Helsinki, and the protocol was approved by the local ethical committee (Ethical Committee Catania 1). All the participants have been given a paper explaining the objectives of the study and have been asked to sign an informed consent prior to be included in the study.

## Results

### Descriptive Analysis

A total of 41 eyes from 21 PD (12 men, 57.1%; mean age 61.5 ± 6.5) and 33 eyes from 17 HCs (9 men, 52.9%; mean age 65.1 ± 10.7) were analyzed. One eye from PD patients and one eye from HCs were excluded due to bad quality of SD-OCT or SD-OCT-A images.

There were no significant differences in age, sex, education, and cognitive status between PD and HCs, as shown in Table 1.

**Table 1:** Demographical and clinical characteristics.

	**PD *n*. 21 (*n*. 41 eyes)**	**Controls *n*. 17 (*n*. 33 eyes)**	***p*-value**
Sex (M)	12 (57.1%)	9 (52.9%)	0.8
Age	61.5 ± 6.5	65.1 ± 10.7	0.2
Age at onset	59.3 ± 7.0	/	
Education	10.5 ± 3.3	11.0 ± 3.6	0.7
MoCA	21.8 ± 5.1	23.4 ± 3.6	0.3
Disease duration (months)	27.4 ± 14.3	/	
HY	1.9 ± 0.4	/	
UPDRS-ME	25.0 ± 6.9	3.2 ± 2.7	**<0.001**
More affected body side			
Left Right Bilateral NA	13 (61.9%) 4 (19.0%) 3 (14.3%) 1 (4.8%)	/ / /	
LD	11 (52.4%)	/	
LED	127.3 ± 142.7	/	
Hypertension	12 (57.1%)	4 (23.53%)	**0.04**

*HY, Hoehn and Yahr scale; UPDRS-ME, Unified Parkinson's Disease Rating Scale part III; MoCA, Montreal Cognitive Assessment; LD, levodopa; LED, levodopa equivalent dose*.

*Bold values are statistically significant p-value (p < 0.05)*.

PD patients were at early stage of disease with a mean disease duration of 27.4 ± 14.3 months (2.3 ± 1.2 years), mean UPDRS-III score of 25.0 ± 6.9, and mean HY score of 1.9 ± 0.4. Ten PD patients (47.6%) were *de novo* patients while 11 were in treatment with dopaminergic drugs. Among the latter, all the patients were in treatment with levodopa, one (9.1%) was in treatment with levodopa and dopamine-agonist (ropinirole), and two (18.2%) were in treatment with levodopa and MAO inhibitors (one with selegiline and one with rasagiline). History of hypertension was more frequent among PD patients compared to HCs, as reported in Table 1.

No deficit in visual acuity, color discrimination, and stereopsis, assessed using validated self-reported questionnaires, were detected among both PD patients and HCs.

### OCT Analysis—Comparison of Retinal Layer Thickness and Peripapillary RNFL Between PD and HCs

The thickness of macular RNFL, GCL, IPL, INL, OPL, and ONL has been found to be significantly lower in PD patients as compared to HCs (Table 2). Close results have been found adjusting by age, sex, and hypertension at multivariate logistic regression analysis, as shown in Table 2.

**Table 2:** Thickness of retinal layers in PD and HC group, assessed by OCT segmentation analysis.

**Layer**	**PD n. 21(n. 41 eyes)**	**Controls n. 17 (n. 33 eyes)**	**Univariate analysis**			**Multivariate analysis**		
			**OR**	***p*-value**	**95% CI**	AdjOR^*^	***p*-value**	**95% CI**
RNFL	13.4 ± 1.9	17.8 ± 2.2	0.36	**<0.001**	0.22–0.56	0.37	**<0.001**	0.23–0.61
GCL	16.1 ± 3.1	21.4 ± 2.2	0.50	**<0.001**	0.37–0.68	0.53	**<0.001**	0.40–0.72
IPL	21.4 ± 2.9	24.2 ± 2.1	0.62	**<0.001**	0.48–0.80	0.63	**0.001**	0.48–0.83
INL	20.7 ± 5.5	28.2 ± 4.5	0.73	**<0.001**	0.63–0.85	0.74	**0.001**	0.63–0.88
OPL	28.5 ± 6.3	31.2 ± 4.8	0.91	0.06	0.83–1.00	0.94	0.23	0.84–1.04
ONL	86.1 ± 12.6	97.7 ± 7.7	0.89	**<0.001**	0.83–0.94	0.88	**<0.001**	0.82–0.94
RPE	15.6 ± 1.6	16.2 ± 1.7	0.78	0.09	0.59–1.04	0.82	0.24	0.59–1.14
ONG	92.8 ± 9.4	101.1 ± 7.9	0.90	**0.001**	0.84–0.96	0.89	**0.001**	0.82–0.95
ON SUP	112.8 ± 16.5	120.6 ± 9.6	0.96	**0.02**	0.92–0.99	0.95	**0.02**	0.91–0.99
ON TEMP	70.5 ± 9.8	78.3 ± 7.8	0.90	**0.001**	0.85–0.96	0.90	**0.004**	0.83–0.97
ON INF	120.2 ± 15.0	119.4 ± 9.2	1.01	0.79	0.97–1.04	/	/	/
ON NAS	73.0 ± 10.2	80.9 ± 8.2	0.91	**0.002**	0.85–0.97	0.93	**0.02**	0.87–0.99

* *For each retinal layer OR are adjusted by age, sex and hypertension. RNFL, Retinal Nerve Fiber Layer; GCL, Ganglionic Cell Layer; IPL, Inner Plexiform Layer; INR, Inner Nuclear Layer; OPL, Outer Plexiform Layer; ONL, Outer Nuclear Layer; RPE, Retinal Pigment Epithelium; ONG, Optic Nerve Global; ON SUP, Optic Nerve–Superior sector; ON TEMP, Optic Nerve–Temporal sector; ON INF, Optic Nerve–Inferior sector; ON NAS, Optic Nerve–Nasal sector*.

*Bold values are statistically significant p-value (p < 0.05)*.

No significant differences in retinal thickness were found comparing PD patients in treatment with dopaminergic drugs and *de novo* PD (Table 3).

**Table 3:** Thickness of retinal layers in PD with and without dopaminergic therapy, assessed by OCT segmentation analysis.

	**PD DT- n.10 (n. 20 eyes)**	**PD DT+ n. (n. 21 eyes)**	***p*-value**
RNFL	13.6 ± 1.6	13.2 ± 2.1	0.5
GCL	16.7 ± 2.1	15.6 ± 3.9	0.3
IPL	21.9 ± 2.2	21 ± 3.5	0.3
INL	20.8 ± 4.2	20.7 ± 6.6	1.0
OPL	29.8 ± 5.2	27.3 ± 7.1	0.2
ONL	84.9 ± 7.8	87.3 ± 16	0.6
RPE	15.6 ± 1.6	15.6 ± 1.7	0.9
ONG	93.3 ± 8.2	92.4 ± 10.6	0.8
ON SUP	114.3 ± 13.5	111.4 ± 19.1	0.6
ON TEMP	72.3 ± 7.7	68.7 ± 11.4	0.2
ON INF	120.9 ± 13.8	119.4 ± 16.4	0.8
ON NAS	73.9 ± 8.7	72.1 ± 11.6	0.6

*DT, dopaminergic therapy; RNFL, retinal nerve fiber layer; GCL, ganglionic cell layer; IPL, inner plexiform layer; INR, inner nuclear layer; OPL, outer plexiform layer; ONL, outer nuclear layer; RPE, retinal pigment epithelium; ONG, optic nerve global; ON SUP, optic nerve—superior sector; ON TEMP, optic nerve—temporal sector; ON INF, optic nerve—inferior sector; ON NAS, optic nerve—nasal sector*.

The overall optic disc and in its superior, temporal, and nasal sectors resulted to be thinner in PD patients' group as compared to HCs (Table 2), also after adjusting for age, sex, and hypertension (Table 2). Similarly to retinal layer thickness, no significant differences in the overall optic disc and in its sectors were found between PD *de novo* and PD treated with dopaminergic drugs (Table 3). Correlation analysis have been performed between clinical and pharmacological scores (MoCA, UPDRS-ME, and LED) and ophthalmological parameters (thickness of each macular retinal layers, overall optic disc, and each sector of optic nerve). There were no significant correlations neither in PD nor in HC group.

### OCT-A Analysis—Comparison of Microvascular Density Between PD and HCs and Association Between Retinal Thickness and Microvascular Pathway

Comparing PD patients and HCs, significant differences in the microvascular density were found neither in the deep capillary plexus nor in the superficial capillary plexus (Table 4).

**Table 4:** Microvascular density pathway between PD and HC, assessed by OCT-A.

	**PD n.21 (n. 41 eyes)**	**Controls n.17 (n. 33 eyes)**	***p*-value**
Foveal thickness	254.3 ± 19.2	259.9 ± 16.8	0.2
SCP whole	44.6 ± 4.4	43.9 ± 3.8	0.5
SCP superior	44.3 ± 4.6	43.5 ± 4.6	0.4
SCP inferior	44.9 ± 4.3	43.7 ± 3.8	0.2
SCP fovea	19.3 ± 5.7	18.4 ± 5.9	0.5
SCP parafovea	47.0 ± 4.5	46.1 ± 4.3	0.4
DCP whole	47.8 ± 4.3	47.8 ± 3.7	0.9
DCP superior	48.0 ± 4.8	48.1 ± 3.9	0.9
DCP inferior	47.6 ± 4.3	47.5 ± 3.8	0.9
DCP fovea	33.8 ± 6.6	32.9 ± 7.9	0.6
DCP parafovea	49.8 ± 4.5	49.9 ± 3.6	0.9
RCP whole	48.5 ± 2.7	48.2 ± 2.1	0.6

*SCP, superficial capillary plexus; DCP, deep capillary plexus*.

**Figure 1:**
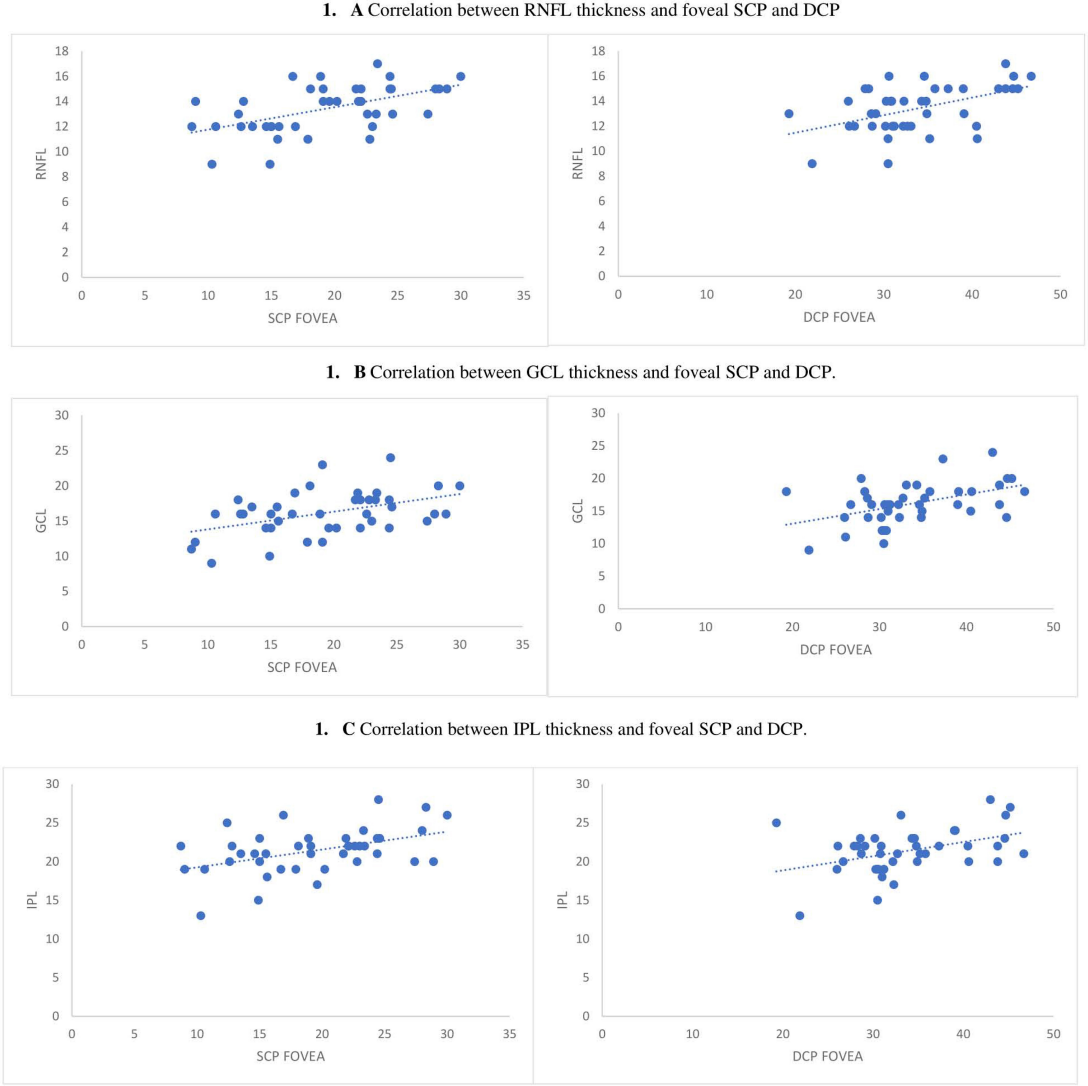
Correlation between RNFL, GCL, and IPL and both foveal deep and superficial capillary plexus. **(A)** RNFL thickness and foveal SCP and DCP. **(B)** Correlation between GCL thickness and foveal SCP and DCP. **(C)** Correlation between IPL thickness and foveal SCP and DCP.

Interestingly, among PD patients, positive correlations were found between the thickness of RNFL, GCL, and INL and the superficial microvascular density (Table 5 and Figure 1).

**Table 5:** Correlation between retinal thickness and microvascular density pathway in the PD group.

	**SCP whole**	**SCP sup**	**SCP inf**	**SCP fovea**	**SCP parafovea**	**DCP whole**	**DCP sup**	**DCP inf**	**DCP fovea**	**DCP parafovea**
**RNFL**										
*r* *p*	**0.32** (0.11) **0.04** (0.15)	**0.33** (0.11) **0.03** (0.13)	0.28 (0.10) 0.08 (0.22)	**0.54 (0.17)** **<0.001 (0.005)**	0.30 (0.09) 0.06 (0.23)	−0.005 0.98	0.12 0.47	−0.15 0.35	**0.49 (0.16)** **0.001 (0.009)**	−0.13 0.42
**GCL**										
*r* *p*	0.25 0.11	**0.32** (0.19) **0.04** (0.10)	0.16 0.31	**0.45 (0.24)** **0.003 (0.02)**	0.18 0.26	0.03 0.85	0.11 0.48	−0.07 0.65	**0.47 (0.20)** **0.002 (0.05)**	−0.05 0.75
**IPL**										
*r* *p*	**0.32** (0.18) **0.04** (0.13)	**0.39 (0.22)** **0.01 (0.05)**	0.23 0.15	**0.45 (0.21)** **0.003 (0.03)**	0.22 0.17	0.003 0.99	0.11 0.49	−0.13 0.43	**0.41 (0.20)** **0.007 (0.03)**	−0.07 0.66

*RNFL, retinal nerve fiber layer; GCL, ganglionic cell layer; IPL, inner plexiform layer; SCP, superficial capillary plexus; DCP, deep capillary plexus*.

*Bold values indicate statistically significant (p < 0.05)*.

Positive correlations between retinal layer thickness and microvascular pathway were confirmed also after adjusting for age, sex, and hypertension, but only in the foveal region (Table 5). Similarly, positive correlations have been found between the RNFL, GCL, and INL thickness and the foveal deep capillary density, confirmed also after adjusting for age, sex, and hypertension (Table 5 and Figure 1). On the contrary, there were no significant correlations between inner retinal layers and retinal microvascular pathway among HCs.

Correlation analysis have been performed between clinical and pharmacological scores (MoCA, UPDRS-ME, and LED) and ophthalmological parameters (superficial microvascular pathway and deep microvascular pathway). There were no significant correlations neither in PD nor in HC group.

## Discussion

Among non-motor symptoms, dysfunction in the visual system has a significant impact on the quality of life of PD patients.

Consistent with previous findings (17–20, 29–31), we found a thinning of peripapillary RNFL and macular RNFL, GCL, IPL, and INL in PD patients as compared to HCs. Interestingly, these findings occur already at the early stage of disease, irrespective of dopaminergic treatment. Furthermore, the main novelty of our study was the positive correlation between RNFL, GCL, and IPL thickness and microvascular density, an association found only in the foveal region of PD patients, an area known to be involved in visual acuity and color discrimination (13).

The mechanisms underlying visual impairment in PD are still unclear (32). It has been supposed that visual defects may be due to abnormalities both in the visual cortex (32) and in the retina. The retina can be easily investigated using OCT, a non-invasive technique that provides high-resolution images of retinal and optic disc anatomy. Several OCT studies reported a peripapillary RNFL thinning in PD patients as compared to HCs (29, 30). Furthermore, several retinal segmentation analysis studies showed a thinning of macular area in PD patients compared to controls, especially in RNFL, GCL, IPL, INL, and OPL (17–20, 31). According to these literature data, we found a reduction in peripapillary RNFL thickness in PD patients compared to HCs, and all retinal layers, except for RPE, were found to be thinner in the macula of PD patients compared to healthy subjects. These findings were independent from age, sex, and especially, history of abnormal blood pressure values and treatment with dopaminergic drugs, suggesting that they occur already in the early phase of PD and that are not influenced by the use of anti-parkinsonian therapies.

Considering each single retinal layer, a stronger association was found between PD and both RNFL and GCL thickness (respectively, OR 0.37 and 0.53, *p*-value < 0.001). These findings could not be surprising considering that retinal dopaminergic cells form synapses with ganglionic cells, that dopamine provides a trophic role on retinal cells, and that RNFL is composed by axonal fibers of ganglionic cells (19). Indeed, although PD is traditionally related to a progressive loss of dopaminergic neurons in the substantia nigra pars compacta and to abnormal alpha-synuclein (αSYN) inclusions, similar histopathological changes have been found elsewhere (8, 9). In fact, physiologically human retina contains dopaminergic neurons (amacrine cells and inner plexiform cells) (33) and a reduction in both dopaminergic cells and dopamine level has been described in PD retina (34–37). Moreover, an association between retinal thinning and striatal dopamine depletion assessed using DATscan has been recently described, suggesting a possible pathologic relationship between retina and brain in PD (38). In addition, neuronal α-synuclein deposits have been proved also in the retina of PD patients. αSYN, as native monomeric inclusions, has been found in the retina of HCs, where it could play a role into neurotransmission, synaptic plasticity, and vesicle trafficking (39). Instead, phosphor-αSYN has been found only in PD patients' retina, just within the somata and neurites of RNFL, GCL, and IPL (40, 41). Thus, it has been supposed that visual impairment reported in PD may be due to dopamine depletion and α-synuclein accumulation in the retinal layers.

Other than neurodegeneration, it has been suggested that vascular degeneration could be an additional risk factor for the onset and progression of PD (42–44). Higher density of string vessel was found in PD patients as compared with unaffected controls, especially in substantia nigra pars compacta (45). Moreover, PD patients showed a higher risk of occurrence of cerebrovascular disease (42) and a higher neuronal vulnerability for ischemic injury has been reported in a PD animal model (46). Accumulation of αSYN along the vessel wall of retinal arteries has been described in a transgenic mouse model of PD (47). Thus, it has been supposed that a vascular retinal pattern could be affected in PD. Performing A-OCT analysis, we found an interesting positive correlation between the thickness of intraretinal layers (RNFL, GCL, and IPL) and both the superficial and deep capillary plexus. This positive correlation was found only in PD patients and only in the foveal region, the area involved in visual acuity and color vision (13). It is well-known that poorly controlled hypertension affects several systems, including retina, playing a central role in the development of microvascular changes and hypertensive retinopathy, but it should be noted that these findings were independent from age, sex, and especially, medical history of hypertension.

Nevertheless, there were no differences in visual acuity, color discrimination, and stereopsis between PD and HCs screened using validated questionnaires. However, the existence of visual impairment in PD patients could not be excluded. Indeed, the sensitivity of self-reported questionnaires for detecting visual defects is low, especially when vision impairment is mild (26–28, 48). Considering that PD patients were at a very early stage, visual defects might be so mild that only instrumental tools could assess abnormal color discrimination, visual acuity, or stereopsis. Nevertheless, a positive correlation between retinal thickness and foveal microvascularization was found only in PD patients and not among HCs. Thus, it could not be excluded that such findings might, at least partly, explain some of the visual disturbances described in PD patients and they could be a starting point for future and more accurate researches.

Remodeling of the fovea has been widely described in PD retina where a thinner and broader foveal pit was found in PD patients as compared to controls (49). Moreover, the inner retinal layer (composed by RNFL, GCL, and IPL) has been reported to be thinner in the perifoveal area of PD patients compared to HCs (20). On the contrary, a correlation between retinal thickness and microvascular pathway was not found in HCs.

To the best of our knowledge, only one study previously investigated the retinal microvascular pathway in PD using A-OCT. Kwapong et al. (21), comparing retinal microvascularization in PD patients and HCs, found a positive correlation only between superficial capillary density and GCL thickness. Moreover, Kwapong et al. (21) described a reduction in retinal microvascular density in PD patients compared with unaffected controls. In our study, there were no differences between the retinal microvascular pathway in PD and HCs, possibly because, even if both studies involved early PD patients, our sample was composed of subjects with a shorter disease duration (2.29 ± 1.19 years vs 3.84 ± 2.80 years, *p*-value 0.02).

Some limitations of our study have to be taken into account when interpreting the results. Sample size is smaller as compared to other studies about this topic, even if it should be noted that OCT findings are in line with literature data (17–20, 29–31). Nevertheless, our sample has been selected applying strict criteria for cases and control, excluding potential confounders that could impact the thinning of the retina. On the other hand, concerning the retinal microvascularization, we cannot entirely rule out that the lack of significant differences between PD patients and HC might be due to a lack of statistical power. A further limit is related to the cross-sectional nature of our study, so it refers to a condition at a specific point in time, and the causality between PD and retina and the temporality between vascular and morphological retinal changes are not known. Moreover, a visual pattern was assessed using only self-reported questionnaires and not instrumental tools that might reveal also subtle visual defects.

On the other hand, one of the main strengths of this study is related to the short disease duration of PD patients. Considering that several studies showed that macular thinning correlates with PD severity (17, 19, 31, 50), our finding of a reduced retinal thickness in early PD patients could suggest the use of this biomarker as a hallmark of brain degeneration. To support our hypothesis, future larger longitudinal studies on progression of retinal thickness along with microvascular abnormalities are surely needed, and it could be interesting to evaluate how fast the changes occur in retinal morphology as compared to in retinal vascularization, and vice versa.

## Conclusion

In conclusion, compared to HCs, the retina resulted to be thinner in PD patients, especially considering RNFL and GCL. Such findings occur already in the early stage of disease and result to be irrespective of dopaminergic treatment. It might be related to a progressive neurodegeneration involving retinal cells, both dopaminergic and not dopaminergic cells, due to αSYN deposition and dopamine depletion. Nevertheless, it cannot be excluded that also microvascular degeneration contributes to the onset or progression of retinal impairment in PD. Indeed, we found a positive correlation between intraretinal layers thickness and capillary density. More interestingly, this correlation was found just inside the foveal region, the area involved in color vision, visual acuity, and contrast sensitivity, all widely reported as impaired in PD. Even if no differences in vision were found between PD and HC groups using validated questionnaires, the presence of visual disturbances so mild to be detected only using instrumental tools cannot be excluded in our PD group. Considering that retinal impairment is already present in the early stage of PD, it could be interesting to evaluate the presence of retinal dysfunction also in the prodromal phase of the disease, when early symptoms or signs of PD neurodegeneration are present, but classic clinical diagnosis is not yet possible.

Hence, further studies are needed to confirm our results, possibly with larger sample size. Nevertheless, considering how easily the retina can be visualized, it is reasonable to hypothesize that the retina might be considered as a biomarker of PD and a useful instrument for monitoring disease progression.

## Data Availability Statement

The datasets generated for this study are available on request to the corresponding author.

## Ethics Statement

The studies involving human participants were reviewed and approved by Ethical Committee Catania 1. The patients/participants provided their written informed consent to participate in this study.

## Author Contributions

AN, MR, and AR: conceptualization. AN and MR: methodology. AN, MR, and CR: formal analysis. AN and CR: investigation. MR, AN, AR, and ALo: resources. NC, CGC, CT, CR, and MF: data curation. AN and CR: writing—original draft preparation. CT, MR, AR, TA, CEC, ALu, MZ, and MF: writing—review and editing. AN and MR: supervision. All authors contributed to the article and approved the submitted version.

## Conflict of Interest

The authors declare that the research was conducted in the absence of any commercial or financial relationships that could be construed as a potential conflict of interest.
